# Epigenetic inactivation of *HOXD10* is associated with human colon cancer via inhibiting the RHOC/AKT/MAPK signaling pathway

**DOI:** 10.1186/s12964-018-0316-0

**Published:** 2019-01-25

**Authors:** Yu-hong Yuan, Han-yu Wang, Yu Lai, Wa Zhong, Wei-ling Liang, Fu-de Yan, Zhong Yu, Jun-kai Chen, Ying Lin

**Affiliations:** 1 Guangdong Provincial Key Laboratory of Malignant Tumor Epigenetics and Gene Regulation, Sun Yat-sen Memorial Hospital, Sun Yat-sen University, No. 107 West Yanjiang Road, Guangzhou, 510120 Guangdong China; 2 Department of Gastroenterology and Hepatology, Sun Yat-sen Memorial Hospital, Sun Yat-sen University, No. 107 West Yanjiang Road, Guangzhou, 510120 Guangdong China; 3 State Key Laboratory of Oncology in South China, Sun Yat-sen University Cancer Center, Guangzhou, 510060 Guangdong China; 4 Department of Radiation Therapy, Sun Yat-sen University Cancer Center, Guangzhou, 510060 Guangdong China; 5 Department of Internal Medicine, Luopu Community Health Service Center of Panyu District, Guangzhou, 511431 Guangdong China

**Keywords:** Colon cancer, Methylation, 5-Aza-dC, *HOXD10*, RHOC/AKT/MAPK pathway

## Abstract

**Background:**

To examine the influence of *HOXD10* on the metabolism and growth of colon carcinoma cells by suppressing the RHOC/AKT/MAPK pathway.

**Methods:**

Thirty-seven paired colon cancer and its adjacent samples from The Cancer Genome Atlas (TCGA) were analyzed. Chip Analysis Methylation Pipeline (ChAMP) analysis was employed for differential methylated points (DMPs) and the differential methylation regions (DMRs) screening. The *HOXD10* mRNA expression and DNA methylation levels were detected by RT-PCR. The Cell proliferation, migration, invasion and apoptosis were respectively measured by MTT assay, transwell assay, wound healing assay and flow cytometry assay in carcinoma cell lines after treated with 5-aza-2′-deoxycytidine (5-Aza-dC) or transfected with HOXD10-expressing plasmid. The expression of *HOXD10* and RHOC was revealed by immunohistochemistry in disparate differentiation colon carcinoma tissues, and the dephosphorylation of AKT and MAPK pathways were detected by RT-PCR and western blot.

**Results:**

The bioinformatics analysis demonstrated that *HOXD10* was hypermethylated and low-expressed in colorectal cancer tissues. The detection of RT-PCR indicated the similar results in colorectal cancer cell lines and tissues. The induction of demethylation was recovered by treatment with 5-Aza-dC and the *HOXD10* in colorectal cancer cell lines was re-expressed by transfection with a *HOXD10* expression vector. The demethylation or overexpression of *HOXD10* suppressed proliferation, migration, invasion and promoted apoptosis in colorectal cancer cells. *HXOD10* suppressed the tumor growth and detected an opposite trend of protein RHOC. AKT and MAPK pathways were notably inactivated after the dephosphorylation due to the overexpression of *HOXD10*.

**Conclusions:**

*HOXD10* was suppressed in colon adenocarcinoma cells, which down-regulated RHOC/AKT/MAPK pathway to enhance colon cancer cells apoptosis and constrain the proliferation, migration and invasion.

## Background

The American Cancer Society (http://www.cancer.org) showed that colorectal cancer was the third leading cause of morbidity and mortality in the United States (US). In addition, the specific incidence rate (SIR) and specific mortality rate (SMR) of CRC was the highest in the World Health Organization (WHO) European region [1]. The most effective therapeutic method for patients with CRC was complete surgical resection, provided the best possibility of long-term survival [2]. It has been illustrated that CRC was still a major health problem in several studies [3–5] and the cancer burden of CRC was anticipated to increase over the next several decades in both developed and developing countries [6]. However, the etiology and underlying molecular mechanisms of colorectal cancer are still unclear.

Epigenetic gene silencing of anti-oncogene through promoter DNA hypermethylation is a common feature in human cancers [7]. DNA methylation refers to the enzymatic addition of a methyl group to the 5 position of cytosine ring by DNA methyltransferase (DNMT) to generate 5-methylcytosine [8], which plays a vital role in the colorectal carcinogenesis [9]. DNMT inhibitor 5-aza-2′-deoxycytidine (5-Aza-dC) is already used in the investigation of many different kinds of cancer, such as gastric cancer [10], breast cancer [11] and urothelial carcinoma [12] on DNA methylation of regulatory elements and gene expression. So far the knowledge of studies which investigate DNA methylation profile in recurrent CRC has been very limited.

HomeoboxD10 (*HOXD10*) is a member of the homeobox gene family, which serves as important transcription factors targeting carcinogenesis-related proteins [13–16]. *HOXD10* is regarded as the primary effector that negatively regulates neoplasm metastasis [17]. Emerging evidence indicates that the expression of *HOXD10* gene is regulated by DNA methylation. In gastric carcinogenesis, *HOXD10* gene is downregulated through promoter hypermethylation [18]. In hepatocellular carcinoma, *HOXD10* gene is silenced by promoter region hypermethylation, which is associated with ERK signaling [19]. The dysregulation of *HOXD10* gene may be involved in diverse pathways that play important roles in tumorigenesis [20]. In malignant breast cancer, loss of *HOXD10* expression during the progression leads to the increment of the pro-metastatic gene RHOC [21]. In gastric tumors, microRNA-10b can promote cell invasion and provoke the up-regulation of RHOC and phosphorylation through targeting *HOXD10* [22]. However, the methylation status of *HOXD10* and mechanism of action in colon cancer with RHOC and AKT pathway are still unclear.

The mitogen-activated protein kinase (MAPK) pathway is a key regulator for apoptosis related to most of the hypermethylated genes while the PI3K/AKT signaling pathway is involved in proliferation process in colorectal cancer [23]. MAPK pathway is over expressed and associated with functional mutation of *HOXD10* gene in human cholangiocellular carcinoma [14] and ovarian cancer [24]. The phosphorylation activation of extracellular signal-regulated kinase (ERK) is a vital regulator for the metastasis and viability of cancer cells [25]. Nevertheless, the underlying molecular mechanisms between the above-mentioned pathways and CRC-associated gene *HOXD10* remain unknown.

This study was designed to confirm the mechanisms and the expression level of *HOXD10* in CRC. We determined *HOXD10* for the follow-up studies*,* which showed hypermethylation and decreased mRNA expression in CRC. 5-Aza-dC treatment can alter the DNA methylation level of *HOXD10*. Our results revealed the overexpression of *HOXD10* had adverse influence on colorectal cancer.

## Methods

### Clinical specimens

For RT-PCR analysis, 15 pairs of frozen colon adenocarcinoma and its adjacent normal tissue specimens were collected from patients with CRC that were diagnosed from 2016 to 2017 at the Department of Gastroenterology and Hepatology, Sun Yat-sen Memorial Hospital. No other therapy, including radiotherapy, chemotherapy was performed prior to entry into the research. Samples used in the study were certified by local ethics committees, and all subjects were given informed consent from patient with available follow-up information.

### Methylome analysis

The colon cancer dataset was obtained from The Cancer Genome Atlas (TCGA) data portal (https://gdc.cancer.gov/). Data for 74 patients were available with complete DNA methylation and were evaluated via the Illumina Infinium Human Methylation 450 BeadArray platform. DNA methylation index (MI) was accounted as β-values. The mean methylated (M) and unmethylated (U) signal intensities for each sample and locus were calculated by the formula (β = M/ [M + U]).

### Demethylation with 5-Aza-dC

5-Aza-2′-deoxycytidine (5-aza-dC) (Sigma-Aldrich, USA) was dissolved in DMSO at 50 mg/ml. Cell lines were plated in 1 × 10^6^ cells/ml for 24 h and treated with 0.5 μM 5-Aza-dC in 0.5% DMSO for 24 h, before growing for 7 days. Cells were harvested for RNA and DNA extraction.

### MS-PCR

Total genomic DNA was extracted by DNA extraction kits (Qiagen, USA) in tissue samples. The DNA content and purity (A260/A280 > 1.8 was considered qualified) were detected by ultraviolet spectrophotometer (Perkin-Elmer, Waltham, MA, USA). The modified DNA was given MSP and non-MSP, respectively (Table 1). The 3% agarose gel was applied to electrophoresis and ethidium bromide in order to stain. R result analysis was detected by gel imaging system (Bio-Rad, Hercules, CA, USA).

**Table 1:** Sequences of MSP primers for qRT-PCR

Name	Sequence
Methylation	
HOXD10 forward	5’ GAATTTGGTAGGTCGAAGGAC 3’
HOXD10 reverse	5’ ATAAACCGCCCTACGAAAAC 3’
No Methylation	
HOXD10 forward	5′ GGAATTTGGTAGGTTGAAGGAT 3’
HOXD10 reverse	5’ AATAAACCACCCTACAAAAAC 3”

### Quantitative real-time RT-PCR (qRT-PCR)

Total RNA (1 lg) was extracted from the CRC cell lines and normal tissue samples using TRIzol reagent (Thermo Fisher Scientific, Waltham, MA, USA). Single-stranded cDNA was subsequently synthesized using an ABI high-profile cDNA synthesis kit (Invitrogen, Carlsbad, CA, USA). The primers were designed by Taqman gene expression assay (Thermo Fisher Scientific, Waltham, MA, USA). The threshold cycle number was automatically determined by ABI 7500 software (Applied Biosystems, CA). The PCR conditions were as follows: 95°Cfor 10 min and then 45 amplification cycles of 94 °C for 20 s, 55 °C for 30 s, and 70 °C for 30 s. The cycle threshold (Ct) values were recorded for both *HOXD10* and *GAPDH*, which was used as an endogenous control. The expression level of *HOXD10* was calculated using theΔCt method. All the samples were assayed in triplicate. The primer sequences for the qRT-PCR are listed in Table 2.

**Table 2:** Sequences of primers for qRT-PCR

Name	Sequence
HOXD10 forward	5’-GACATGGGGACCTATGGAATGC-3’
HOXD10 reverse	5’-TGGTGGTTCACTTCTCTTTTGG-3′.
GAPDH forward	5’-GACCTGACCTGCCGTCTA-3’
GAPDH reverse	5’-AGGAGTGGGTGTCGCTGT-3

### Expression vector for *HOXD10*

Full-length *HOXD10* cDNA (GenBank accession number NM_002148.3) was cloned into the pcDNA3.1 expression vector. Transient transfection was performed using Lipofectamine 3000 (Intrivogen, Carlsbad, CA) according to the manufacturer’s instructions. Empty pcDNA3.1 expression vector was transfected as vector control.

### Western blot

Cell pellets were solubilized in electrophoresis sample buffer, sonicated for 10 s, and boiled for 10 min. The protein concentration of cell lysates was measured, and 3 mg protein was separated by SDS-PAGE, and then transferred to a polyvinylidene fluoride microporous membrane (MILLI-PORE, Billerica, MA). Membranes were blocked in PBS with 5% milk powder and probed with anti-AKT (ab81283, ABcam, USA), anti-ERK (ab54230, ABcam, USA), anti-β-actin (ab11003, ABcam, USA), anti-RHOC (ab64659, ABcam, USA), anti-p-ERK (ab201015, ABcam, USA), ani-p-AKT (ab38449, ABcam, USA), at 4 °C overnight. After washing, the membrane was cultured with HRP-linked goat anti-rabbit Ab (Cell Signaling Technology). Blots were visualized by ECL (ECL Plus; Amersham Pharmacia Biotech, Uppsala, Sweden), on the basis of the manufacturer protocol. Results were captured digitally using a LAS1000 Lumino Image Analyzer (Fuji Photo Film, Tokyo, Japan).

### MTT assay

SW480 and LoVo cells were incubated at 1 × 10^4^ cells/well in 96-well plates. Cells were harvested for 24 h at 37 °C in the presence of 5% CO_2_, before the medium took the place of serum-free medium. The cell lines were treated with 5-Aza-dC (1 μM) at 0 h, 24 h, 48 h, 72 h, 96 h and cell proliferation was detected using the MTT assay. Absorbance at 490 nm was surveyed by a microplate reader (Labcompare, San Diego, CA, USA).

### Wound healing assay

The tumor cell migration capacity was evaluated by the wound-healing assay. In short, 1 × 10^6^ cells were seeded in six-well plates, cultured overnight, and transfected with *HOXD10* expression vector or empty vector. The cell layer was scratched with a sterile plastic tip when the culture had reached approximately 90% confluency, followed by washing with culture medium twice and cultured again for 48 h with serum-reduced medium containing 1% FBS. At different point in time, photographic images of the plates were captured under a microscope and the data were summarized on account of sextuple assays for each experiment.

### Transwell assay

The ability of cell invasion was appraised by 6.5-mm Transwell chambers with a pore size of 8 μm (Corning, USA). A concentration of 50 μg/chamber of a basement membrane pre-coated the Transwell upper chambers in the form of Matrigel (BD Biosciences, USA). Putting RPMI-1640 with 10% FBS into the bottom chamber, before CRC cells (3 × 10^4^/chamber) were seeded in serum-containing media in the upper well of the Transwell chambers. The cells were harvested for 48 h, and then removed those that not yet invade through the pores with the cotton swab. The invasive cells in chamber under the filter surface were immobilized in 70% ethanol, dyed with 0.1 mg/ml crystal violet solution, and counted below a microscope (× 20 magnification). Specific experiments had inserts in triplicate, and five selected fields were counted per insert at random.

### Flow cytometry (FCM) assay

Cell apoptosis was detected by the Annexin V-FITC apoptosis detection kit on the basis of the manufacturer instructions. In brief, a total of 3 × 10^5^ CRC cells and various concentrations (10, 25, 50,100 μmol/L) of emodin were added to the wells after 24 h of incubation at 37 °C. The control group was treated with the equivalent quantity of DMSO (0.2%). Cells of each sample were harvested after an additional 12 or 24 h and suspended in 500 μl of Annexin V binding buffer (1X). Annexin V-FiTC (5 μl) and 5 μl of propidium iodide (PI) were added and incubated for 15 min in dark. The stained cells were evaluated by flow cytometry using a FACS Calibur (BD Biosciences, San Jose, CA, USA).

### Immunohistochemical

After collection of the tumor, 5 μm thin sections of colorectal cancer were immobilized in 4% Paraformaldehyde (PFA) and then embeded in paraffin. Sections were placed on poly L-lysine coated glass slides, immunostained with Envision Flex mini kit (Dako) and in a graded alcohol series to dehydrate and then embed the paraffin. The Anti-Ki67 antibody (Abcam, USA, ab15580) was used to the sections (1:250), and then harvested overnight. The tumor sections were cleaned, which applied (1:500) an anti-Rabbit secondary antibody (Abcam, ab98488).

### Statistical analysis

GraphPad Prism 6.0 Software (GraphPad Inc., La Jolla, CA, USA) was used analysis statistical. All results were repeated for three times. One-way analysis of variance (ANOVA) tested differences of expression among different groups. A figure of *P* < 0.05 was supposed to statistically significant.

## Results

### Distribution of top 1000 differentially methylated imprinted CpG sites

According to CpG island neighborhood (shores, shelves, islands, and opensea), the distribution of top 1000 CpG sites which were differentially methylated demonstrated that the CpG regions based on island of the notably hyper- or hypo- methylated CpG sites were scattered separately.

**Fig. 1:**
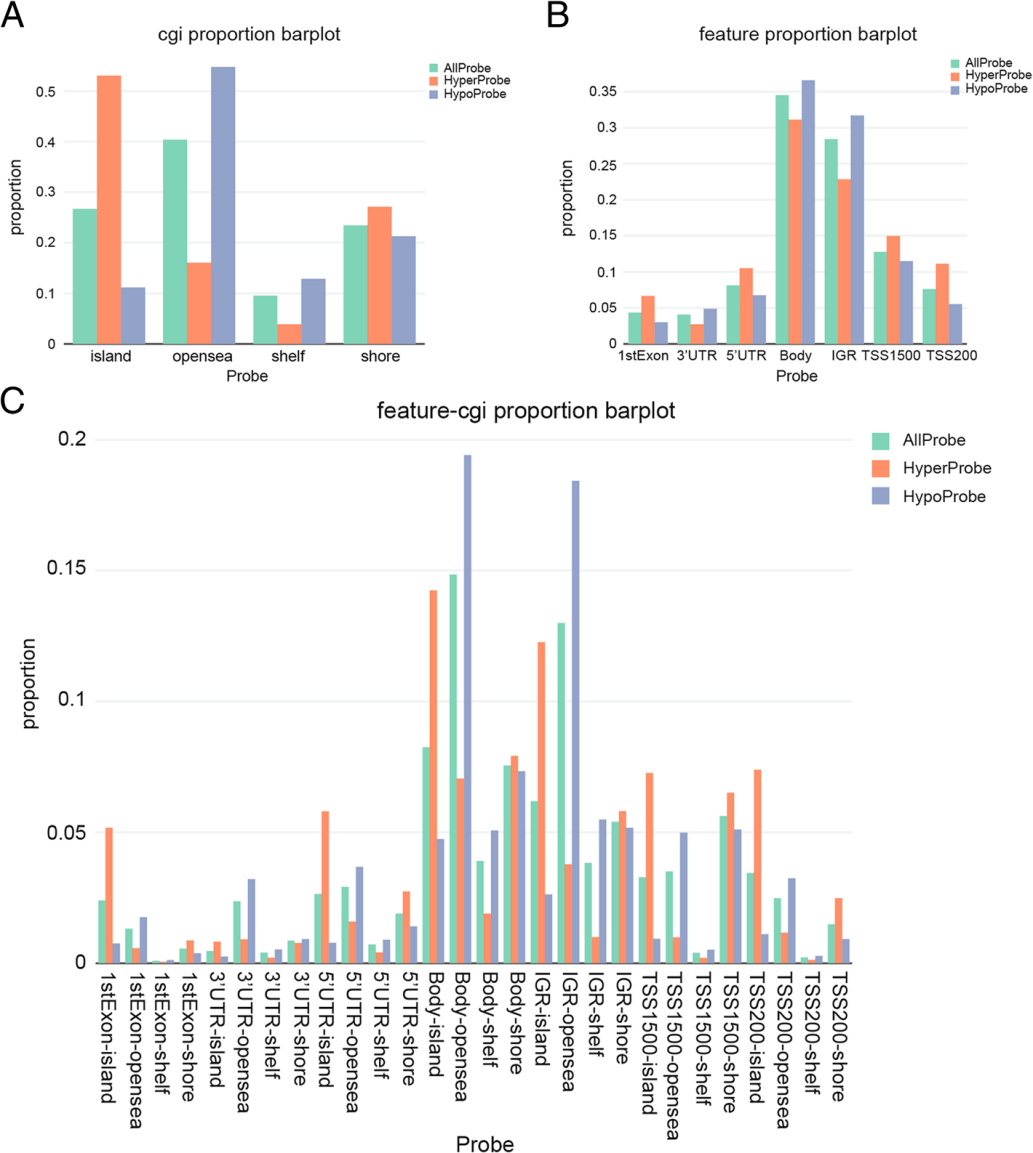
Distribution of top 1000 differentially methylated imprinted CpG sites. **a** Distribution of top 1000 differentially methylated imprinted CpG sites according to CpG islands (shores, shelves, islands, and opensea). **b** Distribution of top 1000 differentially methylated imprinted CpG sites according to the gene position (1stExon, 3’ UTRs or 5’ UTRs, body, IGR, TSS1500 and TSS200). **c** Distribution of top 1000 differentially methylated imprinted CpG sites according to the information of genetic and epigenetic annotation

53% of the hypermethylated CpG sites can be seen in CpG islands. In contrast, shelf had the least CpG methylated sites, and just 4% of the hypermethylated CpG sites were in this region (Fig. 1a). Based on the position relative to genes (1stExon, 3’ UTRs or 5’ UTRs, body, IGR, Transcription Start Sites 1500 bp), the distribution of top 1000 differentially methylated CpG sites illustrated that the highest level of probes was situated in the body (Fig. 1b). The distribution of top 1000 differentially methylated imprinted CpG sites (Fig. 1c) was showed the information of synthesized epigenetic and genetic annotation. All the above, the probes which were differentially methylated were scattered separately primarily in the CpG islands in comparison to the integrated distribution of all probes.

### Significant difference of CpG sites between colon cancer and its adjacent tissues

**Fig. 2:**
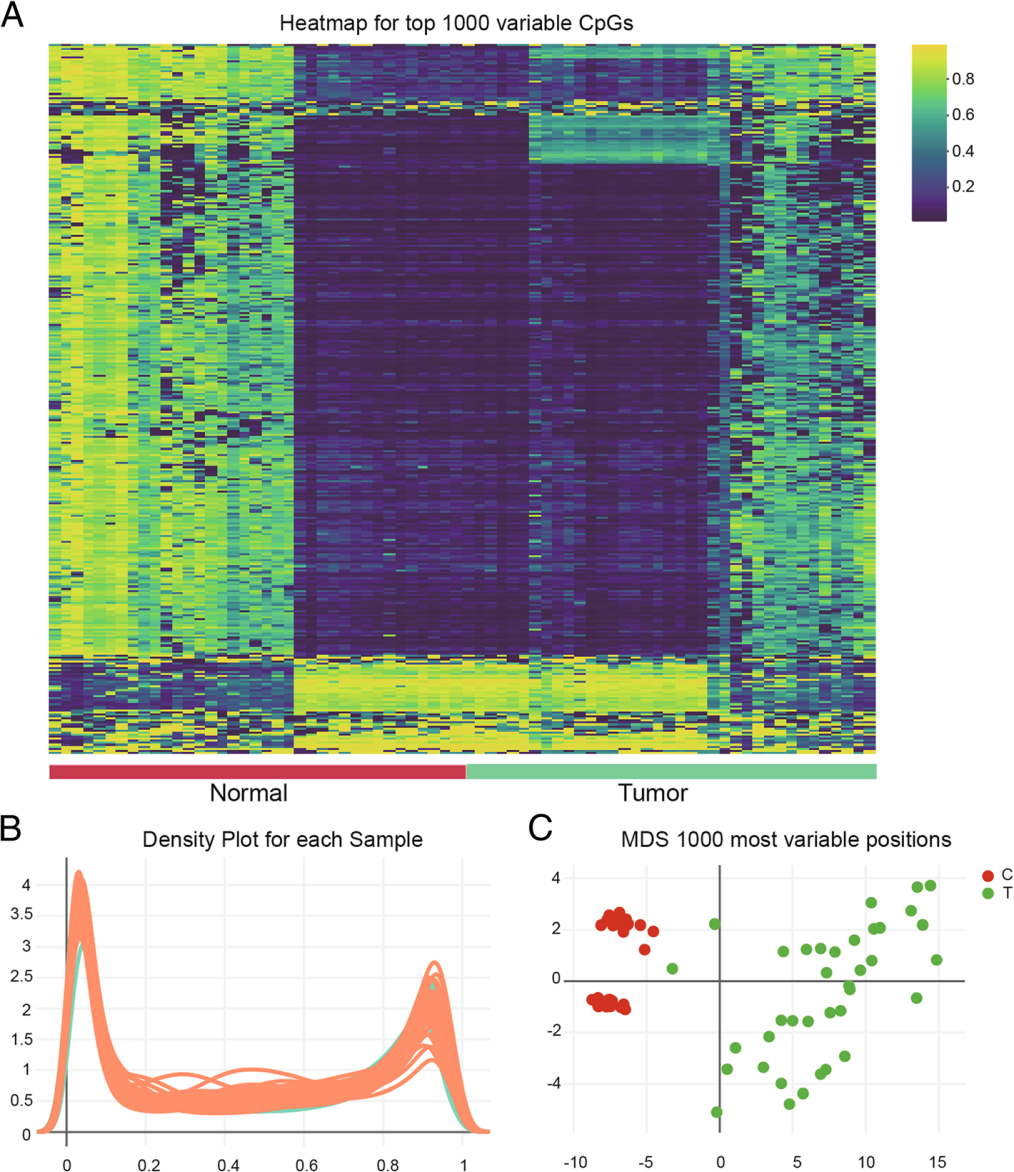
Genome-wide methylation data was from TCGA for 37 available COAD (Colon Adenocarcinoma) tumor/surrounding pairs. **a** Heatmap of top 1000 differentially methylated imprinted CpG sites. **b** Density plot of methylated DNA intensity for each sample. The quality of the data for each specimen was visualized using a density plot, displaying different β-value distributions (0 indicating unmethylated sites, 1 indicating fully methylated sites). **c** Multi-dimensional scaling (MDS) plot showing differential clustering of control vs. tumor tissues

The heatmap of top 1000 differentially methylated imprinted CpG sites illustrated a massive difference of DNA methylation situation between the carcinoma and benign samples (Fig. 2a). In comparison of β-value distributions (0 indicating unmethylated sites, 1 indicating fully methylated sites) density plots from every sample, which were used to analyze poor performing arrays on account of a large deviation from the remaining of the samples (Fig. 2b). Multi-dimensional scaling (MDS) plot indicated variant clustering of normal vs. tumor tissues (Fig. 2c). Overall, we chose quality samples, indicating the significant difference between normal and tumor group.

### *HOXD10* hypermethylation was related to lower mRNA expression in CRC

**Fig. 3:**
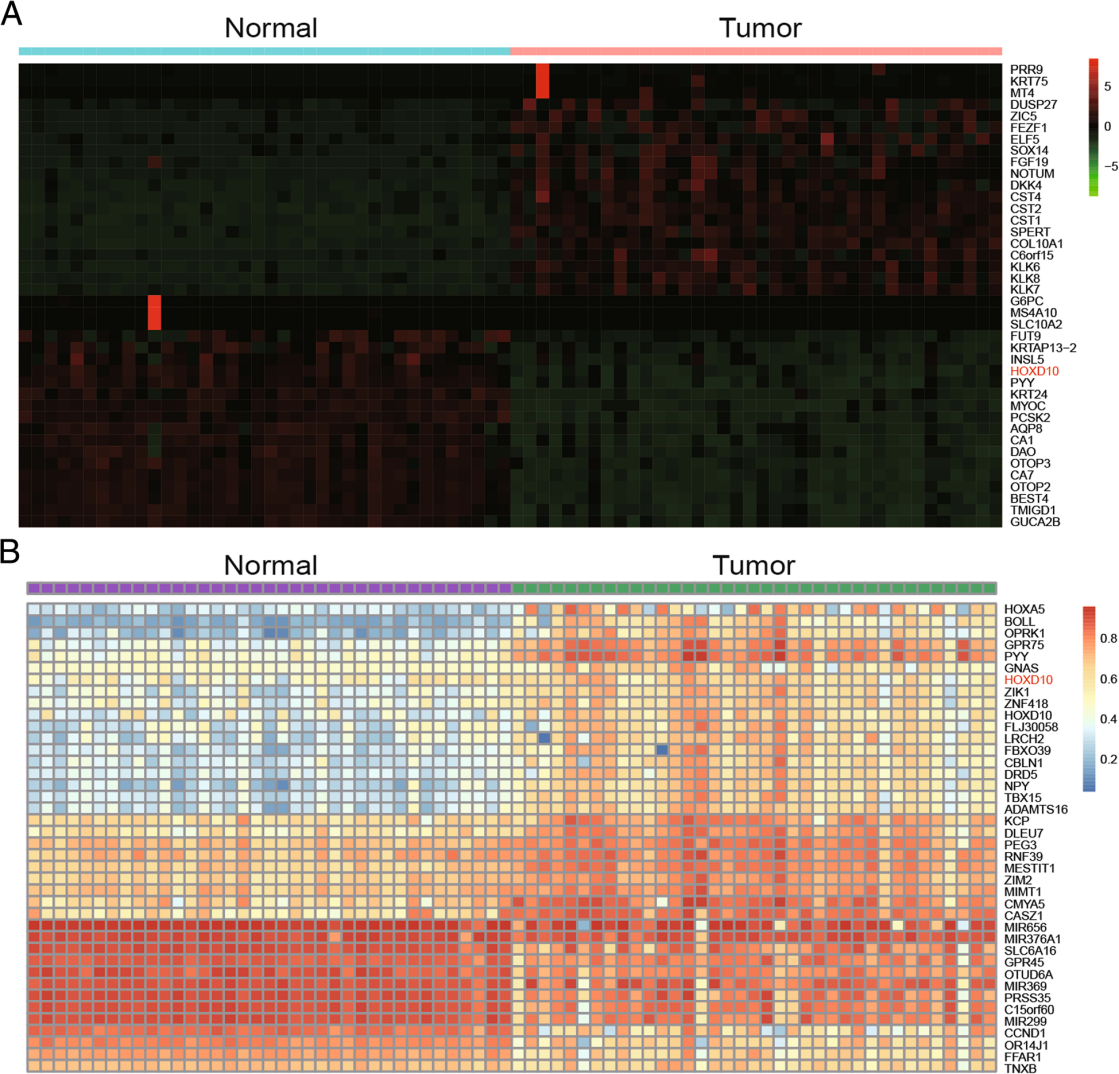
*HOXD10* hypermethylation was associated with lower mRNA expression. **a** Differential mRNA expression analysis using paired tumor/surrounding tissues identified top 40 differentially mRNA genes. Lower *HOXD10* mRNA expression in tumor tissues compare to paired normal tissues. **b** Differential methylation analysis using paired tumor/surrounding tissues identified top 40 differentially methylated genes. Higher *HOXD10* methylation in tumor tissues compare to the counterpart

The heat map of top 40 differentially mRNA expression in tumor and paired surrounding tissues indicated that mRNA expression was lower in colon tumor tissues than the counterpart (Fig. 3a). The top 40 differential methylation genes were analyzed by differential methylation analysis, showing the higher *HOXD10* methylation in carcinoma tissues vs. non-tumor tissues (Fig. 3b). To sum up, the decreased mRNA expression and hyper-methylation of *HOXD10* can be manifested in tumor group.

### *HOXD10* gene was hyper-enriched by CpGs and showed significant difference between normal and tumor group

**Fig. 4:**
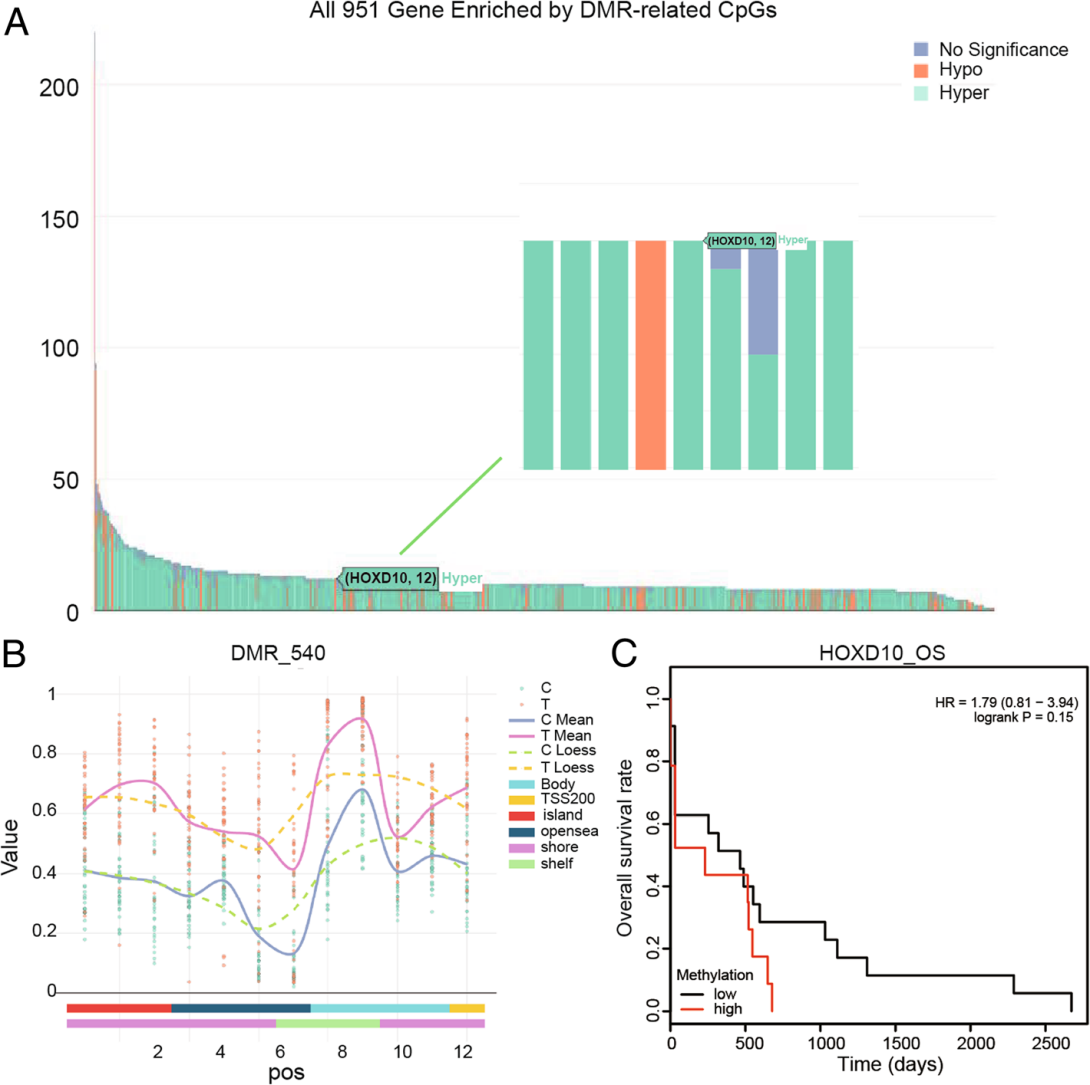
*HOXD10* was enriched by CpGs by DMR (differentially methylated region) analysis. **a** The bar chart shows that *HOXD10* was hyper-enriched by DMR-related CpGs among all 951 genes. **b** A differentially methylated region (DMR_540) of *HOXD10* demonstrates that the expression of this gene in tumor group apparently higher than normal group. **c** Kaplan-Meier plot of the overall survival rate of COAD patients based on *HOXD10* -high or -low methylation

According to the differential methylation region (DMR) analysis, all 951 genes filled with DMR-related CpGs and *HOXD10* were hyper-enriched by CpGs related to DMR (Fig. 4a). The relevant heatmap indicated the normalized methylation data were showed in a blue-red scale from lower to higher methylation. Besides, a differentially methylated region 540(DMR_540) of *HOXD10* indicated the remarkable difference in normal group and carcinoma group which had a higher value than the normal group (Fig. 4b). Although no statistical significance was found, high HOXD10 methylation tended to be associated with poorer prognosis by Kaplan-Meier (logrank *P* = 0.15).The log-rank test was used to calculate *P* values (Fig. 4c).

### Eight CpG sites for *HOXD10* in DMR_540

**Fig. 5:**
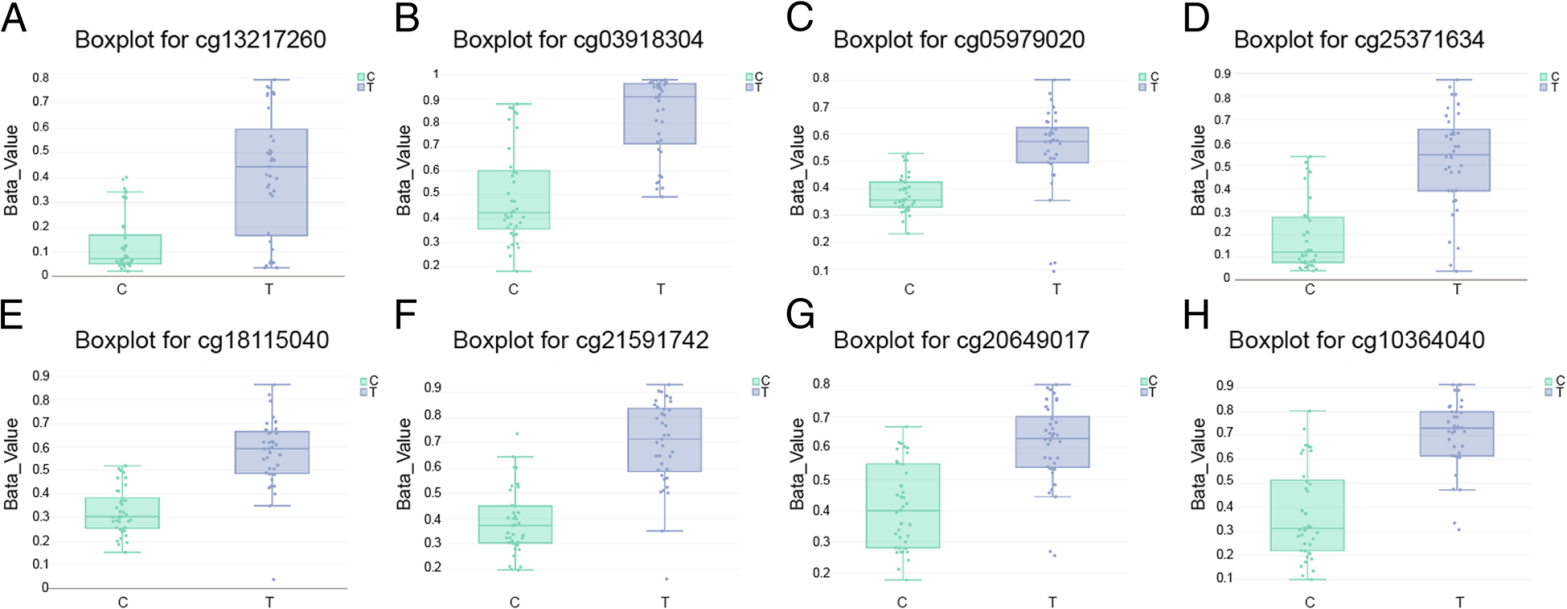
Eight CpG sites for *HOXD10* which presented in boxplot displayed an increased methylation in the tumor group. Boxplot for (**a**) cg 13,217,260, (**b**) cg 03918304, (**c**) cg 05979020, (**d**) cg 25,371,634, (**e**) cg 18,115,040, (**f**) cg 21,591,742, (**g**) cg 20,649,017, (**h**) cg 10,364,040

It has been illustrated that CpG sites with the identical CpG Island tend towards behave in an integrated manner. We used this feature of DNA methylation to study changes in different regions between cancer tissues and adjective normal tissues and recognized DMR_540 for *HOXD10*. Boxplots of methylation data for 8 differentially methylated imprinted sites for *HOXD10* revealed an ascending methylation in the cancer group. Boxplots such as cg 13,217,260, cg 03918304, cg 05979020, cg 25,371,634, cg 18,115,040, cg 21,591,742, cg 20,649,017 and cg 10,364,040 were shown in Fig. 5a-h. To sum up, these results indicated that DNA methylation level of *HOXD10* in CRC was boosted.

### Methylation of *HOXD10* is associated with gene silencing

**Fig. 6:**
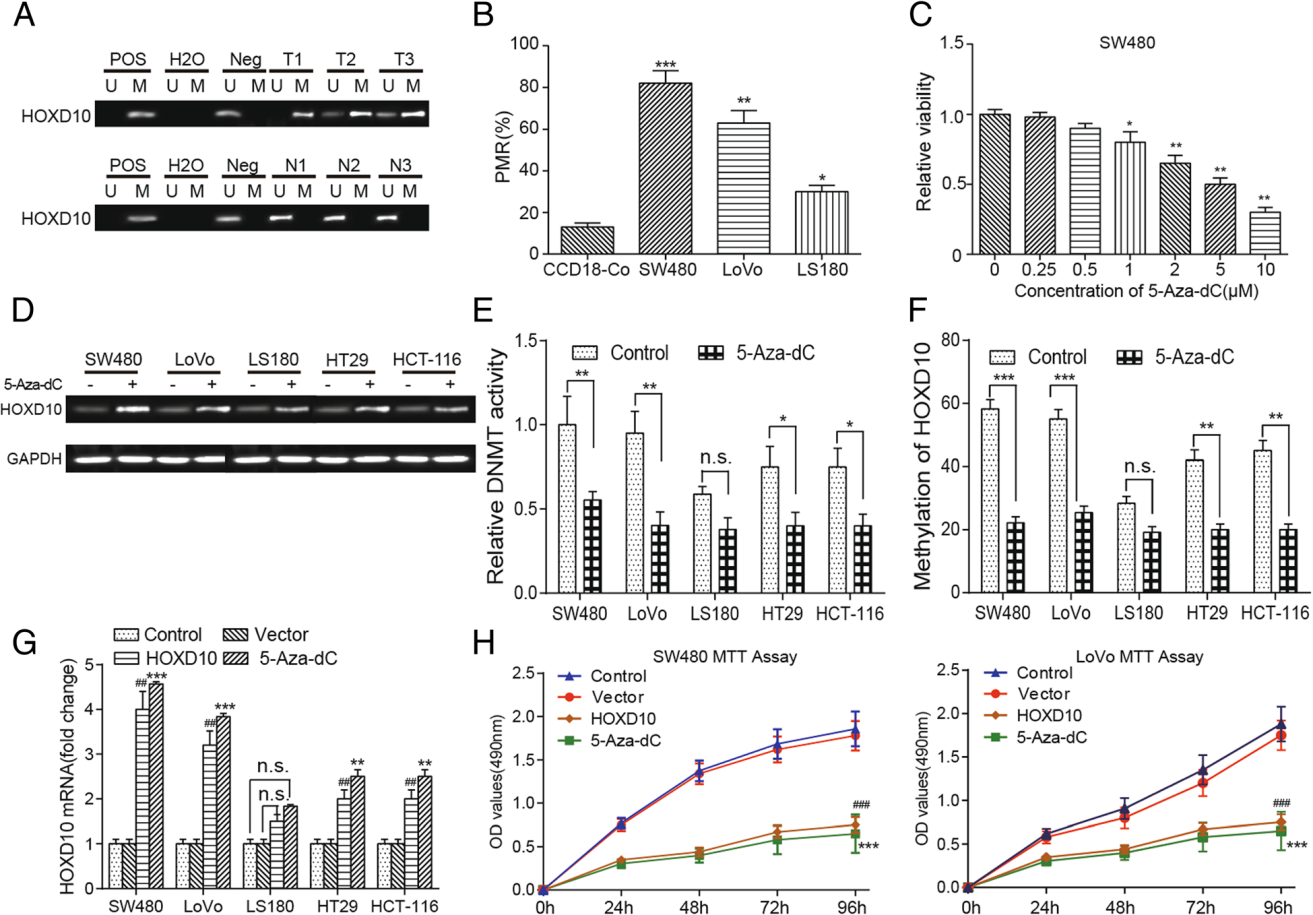
5-Aza-dC induced demethylation and re-expression of silenced *HOXD10*. **a** Higher *HOXD10* methylation in tumor tissues compare to paired normal tissues. ‘Pos’ represented the positive controls for the methylated (M) and unmethylated (U) allele. The normal peripheral lymphocytes DNA was used as negative control. **b** *HOXD10* was confirmed to be hypermethylated in colon adenocarcinoma cell lines SW480 and LoVo compared with normal colon cell line CCD-18Co cell line. The change of DNA methylation level was minimal in LS180 (COAD cell line) compared with CCD-18Co cells. * *P* < 0.05, ** *P* < 0.01, *** *P* < 0.001, compared with the CCD-18Co cell lines. **c** Minimum effective dose of 5-Aza-dC was determined by MTT. 1 μM showed difference. * *P* < 0.05, ** *P* < 0.01, compared with the 0 μM group. **d** The methylation level of *HOXD10* decreased after the treatment with demethylation agent 5-Aza-dC in SW480, LoVo, LS180, HT29 and HCT-116 cell lines. **e** Relative DNMT activity was decreased after treatment of the cells with 5-Aza-dC (1 μM) for 72 h. Compared with the control group, ** *P* < 0.01. **f** 5-Aza-dC (1 μM) treatment decreased *HOXD10* methylation in SW480, LoVo, LS180, HT29 and HCT-116 cell lines. Compared with the control group, *** *P* < 0.001. **g** The mRNA expression of *HOXD10* in SW480, LoVo, LS180, HT29 and HCT-116 cell lines was higher after treatment with 5-Aza-dC (1 μM) for 72 h or overexpression of *HOXD10*. *** indicated *P* < 0.001 compared with the control group; ## indicated *P* < 0.01 compared with the vector congtrol group. **h** Cell proliferation was suppressed in SW480 and LoVo cells after treatment with 5-Aza-dC (1 μM) for 72 h or overexpression of *HOXD10*, determined by MTT assay. Compared with the control group, *** *P* < 0.001. Compared with the vector control group, ###*P* < 0.001

We analyzed the *HOXD10* methylation levels in 15 CRC and 15 para-carcinoma tissue. Constant methylation was observed in CRC, but less in paired non-cancer tissues,the typical result can be seen in Fig. 6a. ‘Pos’ represented the positive controls for the methylated (M) and unmethylated (U) allele. The normal peripheral lymphocytes DNA which served as negative control were included for each PCR.*HOXD10* were confirmed hypermethylation in colon cancer cell lines SW480,LOVO and LS180 compared with normal colon cell line CCD18-Co (Fig. 6b). MTT assay determined the minimum effective dose of 5-Aza-dC, and showed difference at 1 μM, which was selected to conduct following experiments (Fig. 6c). *HOXD10* expression was determined by RT-PCR, indicating that the methylation level of *HOXD10* decreased after 5-Aza-dC treatment in SW480, LoVo, HT29 and HCT-116 cell lines while LS180 cell line showed little change (Fig. 6d). DNA methylation can be efficiently induced by DNA methyltransferase, whose activity was restrained by 1 μM 5-Aza-dC (Fig. 6e). The demethylation of *HOXD10* after treatment with 1 μM demethylation agent for 72 h in colon carcinoma was analyzed using RT-PCR. 5-Aza-dC (1 μM) treatment decreased *HOXD10* methylation in SW480, LoVo, LS180, HT29 and HCT-119 cell lines (Fig. 6f). To investigate the function of *HOXD10* in colorectal tumors, we transfected CRC cells with a *HOXD10* vector or empty vector and then confirmed the level of mRNA. The empty pcDNA3.1 expression vector group (Vector) was used as negative control versus HOXD10-expressing vector group and the control group with no treatment or transfection was used as blank control. The mRNA expression of *HOXD10* in SW480, LoVo and LS180 cell lines was higher after treatment with 5-Aza-dC (1 μM) for 72 h, which was similar to the overexpression of *HOXD10*. (Fig. 6g). In conclusion, 1 μM 5-Aza-dC were chosen in the following experiments as demethylation agents, and the demethylation and mRNA expression of *HOXD10* increased after the treatment of 5-Aza-dC or overexpression of *HOXD10*.

### *HOXD10* inhibited CRC cell proliferation, cell function and induced apoptosis

**Fig. 7:**
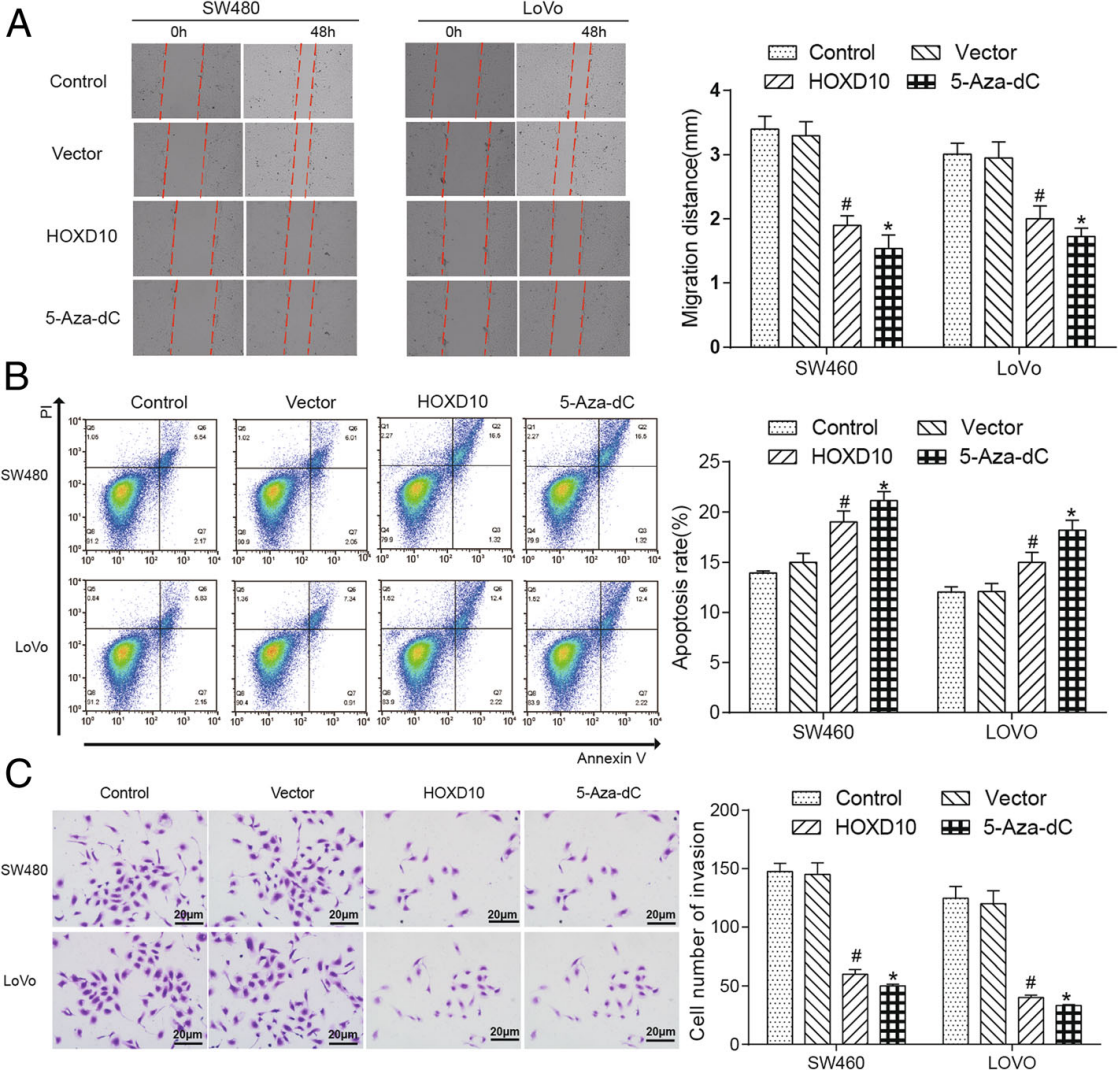
*HOXD10* re-expression inhibited cell migration, invasion and promoted the cell apoptosis. **a** *HOXD10* reduced the migration rates of SW480 and LoVo cells in scratch wound-healing assay, and photographs were taken at 0, 48 h after the wound was made (left). Statistical plot of the average number of migrated SW480 and LoVo cells in each group (right). * indicated *P* < 0.05 in comparison with the control group and # indicated *P* < 0.05 in comparison with the vector control group. **b** The result of flow cytometry showed *HOXD10* overexpression induced SW480 and LoVo cells apoptosis (left). Statistical plot displayed percentages of apoptosis in SW480 and LoVo cells (right), *P < 0.05;#*P* < 0.05. **c** Overexpression of *HOXD10* significantly decreased the invasive potential of both SW480 and LoVo cell lines through Matrigel invasion Transwell assay (left). Statistical plot of the average number of invaded SW480 and LoVo cells in each group (right). The graph showed the mean ± SD. *P < 0.05; #*P* < 0.05

The cell proliferation was restrained after demethylating or overexpress the *HOXD10* by seeding SW480 and LoVo cell lines for MTT assay (Fig. 6h). The cell migration ability in tumor cell lines, which determined by Wound Healing assay, was inhibited after the treatment of 5-Aza-dC or overexpression of *HOXD10* from Fig. 7a, left, which captured at 0 and 48 h after the wound was made, and corresponding histograms showed the same trend (Fig. 7a, right). The cell apoptosis rate in SW480 and LoVo cells was remarkable increased by flow cytometry analysis (Fig. 7b, left), and the related histogram showed the evident increment of apoptosis rate (%) (Fig. 7b, right). And CRC cell invasion ability was dramatically suppressed by overexpression of *HOXD10* in SW480 and LoVo cells from the figure of flow cytometry assay (Fig. 7c). Based on the above observations, it was probably that *HOXD10* functioned as a cancer suppressor by restraining the cell proliferation, migration, invasion and promoting the cell apoptosis in tumorigenesis of the colon.

### The expression of *HOXD10* displayed contrary tendency with malignant neoplasms gene RHOC which related to AKT/ERK pathway activities

**Fig. 8:**
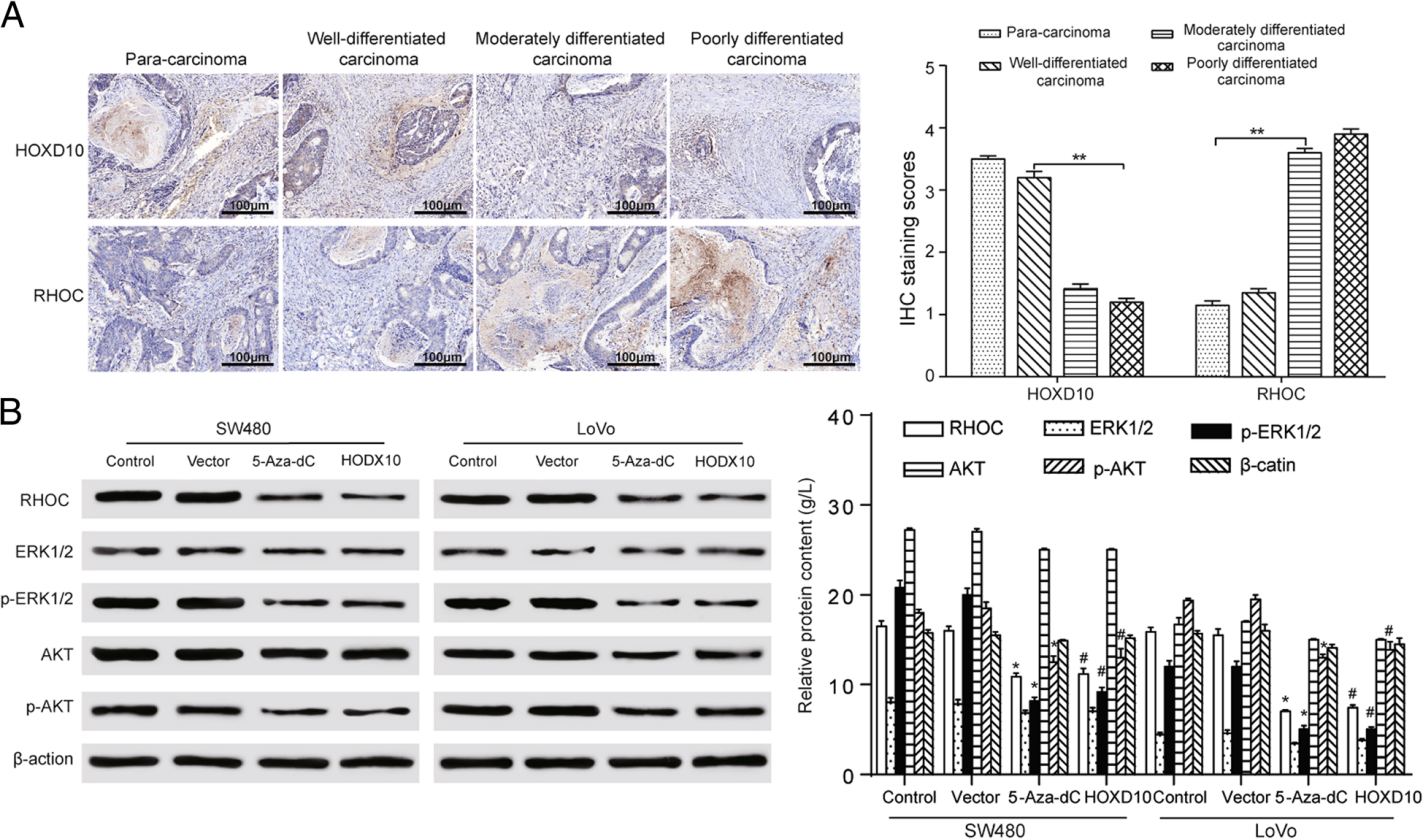
The expression of *HOXD10* displayed contrary tendency with protein RHOC. **a** *HOXD10* stainings were gradually weakening while RHOC expression showed a reverse trend in para-carcinoma (× 100), well- (× 400), moderately- (× 400) and poorly-differentiated carcinoma (× 400), observed by immunohistochemistry (left). Statistical plot illustrates IHC staining scores in SW480 and LoVo cells (right). **b** Suppressed protein expression levels of RHOC, phosphorylated AKT, and phosphorylated ERK (1/2), and unchanged expression of AKT and ERK (1/2) in SW480 and LoVo cells compared with the control groups (left). Statistical plot showed the relative protein expression in RHOC/AKT/ERK in SW480 and LoVo cells (right). *P < 0.05, **P < 0.01 compared with the control group; #*P* < 0.05, ##*P* < 0.01 compared with the vector control group

*HOXD10* protein level was evaluated in carcinoma or para-carcinoma tissue from patients with CRC via immunohistochemistry (IHC) in order to investigate how *HOXD10* had an effect on CRC. Lower *HOXD10* and higher RHOC expression can be seen in poorly-differentiated CRC tissues form the result of immunohistochemical stainings. The *HOXD10* staining was primarily positioned in the cell nucleus, however, the RHOC staining was localized in the cell cytoplasm. The *HOXD10* stainings are strong, strong, moderate and weak in para-carcinoma (× 100), well- (× 400), moderately- (× 400) and poorly-differentiated carcinoma (× 400), respectively, while the RHOC stainings are weak, weak, moderate and strong in the same carcinoma groups, respectively. (Fig. 8a, left) Statistical plot illustrated IHC staining scores in different stages of carcinoma (Fig. 8a, right). The RHOC protein levels showed a significant decrease in the *HOXD10* overexpressed group and 5-Aza-dC group. The up-regulation of *HOXD10* resulted in AKT and ERK/MAPK dephosphorylation, demonstrating that the AKT and ERK/MAPK pathways were markedly inactivated (Fig. 8b, left). Statistical plot showed the relative protein expression in RHOC/AKT/ERK in SW480 and LoVo cells (Fig. 8b, right). The results turned out that RHOC was a pivotal negative mediator of CRC development depending on *HOXD10* and also a gene that the AKT and ERK/MAPK pathways were involved in.

## Discussion

In this research, we had uncovered that *HOXD10* played a vital role in the progression of the colorectal cancer. The evidence has been provided *HOXD10* showed hypermethylation and decreased mRNA expression in CRC. Also, we focused on analyzing the pro-metastatic gene RHOC and the phosphorylation activation of the AKT and MAPK signaling pathways altered after *HOXD10* demethylation. Exploring the role of anti-oncogenes *HOXD10* in human colorectal would lead to a deeper understanding of the underlying mechanism of CRC, as well as stimulate the development of new treatment strategies.

DNA methylation could cause transcriptional silencing of anti-oncogene, which was a common epigenetic modification. Existing research indicated that the methylation of cancer suppressor genes was in close relationship with cancer development [26]. Growing evidence showed clearly that the CpG island hypermethylation in oncogenes played a fundamental role in the process of colon cancer development [27, 28]. Previous studies have showed that *HOXD10* which was aberrantly hypermethylated in papillary thyroid cancer may act as a tumor suppressor [29]. *HOXD10* was upregulated in 82 (89.13%) normal gastric mucosa samples and in 242 of 436 (55.5%) cases of gastric cancer [30]. Lin et al. showed that of promoter hypermethylation *HOXD10* combined with *ZIC1* and *RUNX3* might be a potential early detection of gastric cancer and precancerous lesions [31]. Daugaard et al. demonstrated that homeobox gene family including *HOXD10*, *HOXD3*, *HOXB3/HOXB4* have potential as biomarkers in lung adenocarcinoma [32]. Thus, *HOXD10* was aberrantly hypermethylated in various carcinoma, which showed the potential of it as biomarker or therapeutic target. Similarly, our study illustrated the *HOXD10* hypermethylation and low mRNA expression in colorectal carcinoma tissues and cells.

5-aza-2′-deoxycytidine (5-Aza-dC) can reactivate epigenetically silenced genes, which was able to inhibit DNA methylation [33]. Besides, 5-Aza-dC is the clinically approved DNA methyltransferase inhibitors (DNMTi) [34]. It is reported that 5-Aza-dC was used in colorectal carcinoma cell lines for *APC* gene, which methylation promoted the migration and proliferation abilities [26]. Moreover, after the treatment with a DNA methyltransferase inhibitor 5-Aza-2′-deoxycytidine, the methylation degree of *HOXD10*, which was correlated with decreased transcript expression, was restored in oral cancer cell lines [35]. Our study has illuminated that *HOXD10* might function as a vital effector that negatively regulated the metastasis and development in oncogenesis of the colon after the treatment of 5-Aza-dC.

Fassan M et al. has proved that a weak cytoplasmic *HOXD10* IHC cytoplasmic positivity with membranous reinforcement can be seen in normal samples while 80% breast cancer tissue samples indicated moderate to strong positivity cytoplasmic *HOXD10* IHC positivity [36]. The result of IHC indicated that positive rate of *HOXD10* protein expression dropped gradually with the increment of histological grade in CRC patients, which was closely connected with a higher expression of pro-metastatic gene RHOC, a gene related to the AKT and MAPK pathway. The same result can be seen in human cholangiocellular carcinoma, the *HOXD10* expression was pretty high in well-differentiated cancerous tissues (25/28, 89.3%), while the *HOXD10* was lowly expressed in well-differentiated cancerous tissues (25/28, 89.3%) [14]. It has been demonstrated that abnormally activated AKT and MAPK pathway is closely associated with growth and metastasis of CRC [37–40]. Previous research suggested that inactivated MAPK/ERK and PI3K/AKT signaling pathways resulted from decreasing the phosphorylation of ERK1/2 and AKT, which were classical signal transduction pathways and played a significant role in colorectal carcinoma progression [41].

## Conclusions

In conclusion, the high methylation and low relative mRNA expression of *HOXD10* in CRC tissue microarray data, cell lines, and tumor tissue samples, combined with the loss of function associated with the AKT/MAPK signaling pathway for the role as anti-oncogenes in the pathogenesis of CRC. Follow-up studies are needed to completely evaluate *HOXD10* in colorectal carcinoma, thus make detailed risk analysis and prognosis easier. Our findings gave an in-depth understanding of *HOXD10* methylation involved in the development of colorectal cancer cell metastasis, which may serve as a possible target for therapy in times to come.

## Abbreviations

ChAMP: Chip Analysis Methylation Pipeline
Ct: Cycle threshold
DMPs: Differential methylated points
DMRs: Differential methylation regions
DNMTi: DNA methyltransferase inhibitors
ERK: Extracellular signal-regulated kinase
HOXD10: HomeoboxD10
IHC: Immunohistochemistry
M: Methylated
MAPK: Mitogen-activated protein kinase
MI: Methylation index
PFA: Paraformaldehyde
PI: Propidium iodide
SMR: Specific mortality rate
SIR: Specific incidence rate
TCGA: The Cancer Genome Atlas
U: Unmethylated
